# Diagnostic Challenge of Extranodal Marginal Zone Lymphoma of Extraocular Muscles: A Case Report

**DOI:** 10.3390/reports9010055

**Published:** 2026-02-09

**Authors:** Vaia-Aikaterini Alexoudi, Dimitris Tatsis, Christos Varelas, Konstantinos Vaxtsevanos, Aggeliki Cheva

**Affiliations:** 1 Department of Oral and Maxillofacial Surgery, Aristotle University of Thessaloniki, 54124 Thessaloniki, Greece; dtatsis@outlook.com (D.T.);; 2 Specialized Cancer Treatment and Reconstruction Centre, “George Papanikolaou” General Hospital of Thessaloniki, Exochi, 57010 Thessaloniki, Greece; 3 Hematology & HCT Unit, George Papanikolaou Hospital, Exochi, 57010 Thessaloniki, Greece; 4 Pathology Department, Faculty of Medicine, Aristotle University of Thessaloniki, 54124 Thessaloniki, Greece; antacheva@yahoo.gr

**Keywords:** extranodal marginal zone lymphoma, extraocular muscle, orbital lymphoma, navigation system, MALT lymphoma

## Abstract

**Background and clinical significance:** The current report examines an unusual case of extranodal marginal zone lymphoma (EMZL) affecting only the extraocular muscles. The diagnostic challenges connected to such atypical manifestations are highlighted. The resemblance to inflammatory or infiltrative processes may lead to diagnostic delays; therefore, therapy administration may be withheld. **Case presentation:** A 77-year-old male was admitted to our hospital with periorbital edema accompanied with vision abnormalities affecting his left eye. The complete diagnostic workup and surgical treatment is presented using a navigation system. Adequate biopsy samples from the delicate orbital tissue can be obtained by utilizing contemporary visualization techniques, particularly navigation systems. The pathology report proved indispensable, with ample raw muscle tissue providing sufficient material from whicha definitive diagnosis was made. The indicated therapy was administered without delay. **Conclusions:** The uncommon, isolated affection of extraocular muscles by extranodal marginal zone lymphoma is exemplified in this case, highlighting the need for early clinical suspicion in order to differentiate this condition from other more prevalent inflammatory pathologies. The implementation of advanced technologies, such as image-guided navigation systems, alongside a highly skilled multidisciplinary medical team, ensures optimal patient results.

## 1. Introduction and Clinical Significance

Extranodal marginal zone lymphomas (EMZLs) consist of a subdivision of B-cell lymphomas that generally occur locally, affecting various extranodal sites that are mainly stimulated by chronic inflammation. In addition, the implication of autoimmune mechanisms in the pathogenesis of the ailment has been proposed [1]. They are also referred to as MALT lymphomas. The most frequent site of MALT lymphoma diagnosis is the gastrointestinal tract, and the pathogenesis mainly involves chronic inflammation induced by Helicobacter pyloripositive gastritis [2,3].

Although gastric MALT lymphoma is the most prevalent, this type of lymphoma can also affect other mucosal areas such as the lungs, salivary glands, thyroid, skin, and periorbital areas [1].

Mucosa-associated lymphoid tissue (MALT) lymphoma prevails among extraocular muscle lymphomas (EOMLs), which are rare, comprising <0.2% to 9.6% of orbital lymphomas [4]. Orbital lymphomas account for approximately 1% of non-Hodgkin lymphomas and 10% of adult orbital malignancies. Orbital/ocular adnexal MALT lymphoma constitutes 50–70% of such cases in Western populations and 80–90% in Asian cohorts, with isolated EOM involvement uncommon (17.6% in select series). EOMLs are predominantly unilateral, with bilateral involvement in 9–12% of cases [5]. Commonly affected muscles include the rectus group (up to 73%), obliques (17%), and levator palpebrae superioris (11%), with an average presentation age of 57–65 years. Physically, orbital MALT lymphomas often present as a localized mass affecting the conjunctiva, the eyelids, the lacrimal apparatus, and, most rarely, the extraocular muscles [6]. Chlamydia psittaci infection is recognized as an etiological factor for orbital MALT lymphoma, often through chronic inflammation induction in periorbital tissues [7].

Diagnosis of orbital/ocular adnexal lymphoma presents a challenge, which requires, first, a high clinical suspicion, and second, an excellent collaboration with the pathologist receiving the pathology specimens. This is due to the limited sample for diagnosis because of both anatomic considerations and the need to delicately manage the specimens.

This case report presents a case of orbital EMZL-MALT lymphoma that represented a diagnostic challenge due to impairment only of the extraocular muscles of the left eye.

## 2. Case Presentation

**Figure 1:**
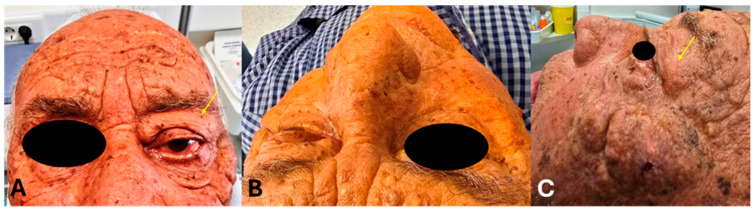
Clinical photographs of the patient: (**A**) anterior, (**B**) superior, and (**C**) lateral perspectives. Periorbital edema is localized on the left-hand side (arrow).

A 77-year-old male presented in the outpatient clinic of the Oral and Maxillofacial Surgery department of General Hospital G. Papanikolaou, Thessaloniki, Greece. The main complaints of the patient included diplopia and periorbital edema localized in the left eye. The patient reported that the symptoms initially occurred approximately 5 months ago but gradually progressed over the past 3 months, at which point he sought medical advice. Progressive eye movement impairment, particularly in the vertical direction, was reported by the patient. The swelling was characterized as non-painful, and the patient also reported a persistent feeling of pressure behind the left eye (Figure 1). There was no fever, and there were no signs of systemic disease. Thyroid hormones and C-reactive protein were both in the normal range. Subclasses of IgG antibodies were not assessed. Additionally, there was no history of trauma, as well as no prior history of ocular disorders or surgeries. Concerning medical history, the patient experienced hypertension and hyperlipidemia, and there was no ocular or neurological history.

During the physical examination, restricted eye movements in all directions—with generalized swelling and edema of the extraocular muscles—were observed. There was a bulging of the left eye, accompanied by proptosis and conjunctival edema (Figure 1).

Ophthalmological assessment was conducted, revealing visual acuity of 9/10 in the right eye and 3/10 in the left eye with correction. Corneal reflexes were normal on direct and indirect testing. Relative afferent pupillary defect was negative. Ishihara color perception was 25/25 in both eyes. The left eye showed anterior segment conjunctival hyperemia. Fundus examination under drug-induced mydriasis demonstrated optic disks with clear boundaries in both eyes.

**Figure 2:**
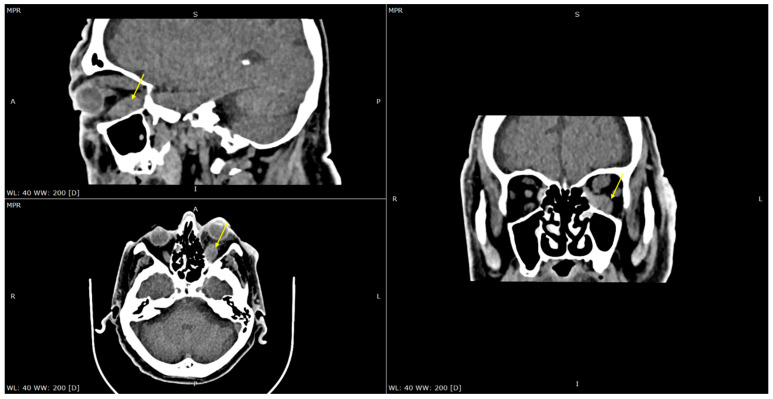
Multiplanar reconstruction of computed tomography. Generalized thickening and edema of all the extraocular muscles, mostly evident in the lower rectus muscle (arrow).

**Figure 3:**
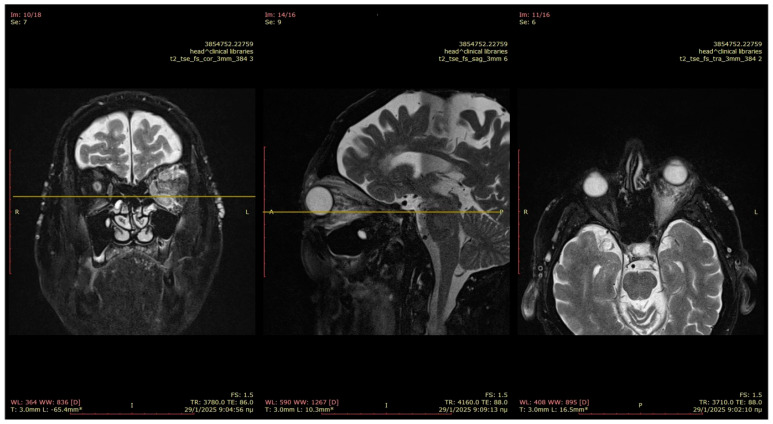
MRI, T2-weighted images from coronal, sagittal, and axial perspectives. Generalized thickening and edema of all the extraocular muscles, mostly evident in the lower rectus muscle.

A contrast-enhanced CT scan and MRI were performed, both indicating generalized thickening and edema of all the extraocular muscles, mostly evident in the lower rectus muscle (Figure 2 and Figure 3). The edema also involved the intraconal and extraconal orbital fat sparing the tendons and the lacrimal gland apparatus. There were no signs of orbital space occupying lesions, and no abnormalities of the optic chiasm were reported. Differential diagnosis was conducted based on the imaging and included extraocular inflammation, orbital myositis, IgG4-related disease, thyroid eye disease, sarcoidosis, and tumor infiltrative disease [8,9]. Thus, further diagnostic work-up was required.

A biopsy of the lower rectus muscle was performed with the use of an image-guided navigation system. Several tissue samples were obtained: one fresh biopsy and the others fixed in formalin for histopathological examination. The biopsy was guided by the navigational system to ensure accurate localization and minimize risk during the procedure.

**Figure 4:**
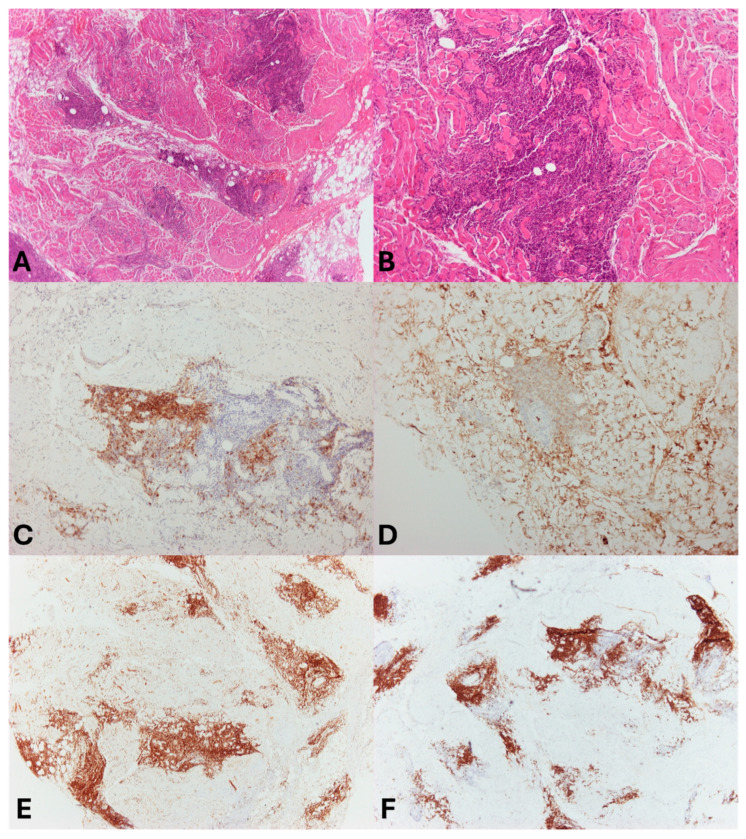
Pathology stains. (**A**) Hematoxylin–eosin (×40). (**B**) Hematoxylin–eosin (×100). (**C**) CD23 (×100) focal positivity. (**D**) IgM (×100) focal positivity. (**E**) CD20 (×40), significant for diagnosis of B-cell neoplasms. (**F**) BCL2 (×40) focal positivity.

Histopathological examination of the specimens revealed extranodal marginal zone lymphoma of mucosa-associated lymphoid tissue (EMZL-MALT), a subtype of non-Hodgkin lymphoma (Figure 4). The tissue demonstrated lymphoid infiltrates composed of small, mature lymphocytes exhibiting marginal zone differentiation. Initially, frozen sections were prepared from the fresh muscle biopsy material. Upon identification of lymphoma, the tissue was embedded in paraffin to obtain formalin-fixed, paraffin-embedded (FFPE) blocks for further immunohistochemical analysis. Hematoxylin and eosin staining highlighted neoplastic lymphoid cells with small, homogeneous, hyperchromatic nuclei. The immunohistochemical staining was CD20+, BCL2+, IgM++/-, CD23+/-, CD3-, CD5-, CD21-, LEF1-, cyclinD1-, BCL6-, CD10-, AnnexinA1-, and T-lymphocyte infiltration. The absence of amyloidosis was indicated by negative Congo red staining. CD5- and cyclinD1- helped exclude mantle cell lymphoma, while CD10-, CD5-, BCL6-, and LEF1- suggested the absence of follicular lymphoma and chronic lymphocytic leukemia/small lymphocytic lymphoma, respectively [5,10,11]. Ki67 was <10%, indicating a low proliferative index consistent with MALT lymphoma. AnnexinA1- assisted in ruling out hairy cell leukemia. No flow cytometry was conducted due to the fact that cell architecture and morphology were well-preserved in the biopsy samples, allowing for a definitive diagnosis based on histopathological and immunohistochemical analyses.

The patient was handed over to the hematology–oncology team to continue treatment. Notably, involvement was confined to the extraocular muscle tissue only, as further investigations did not provide evidence of systemic involvement. The diagnosis was established solely on the basis of muscle biopsy.

Initial staging investigations, including bone marrow biopsy, computed tomography, and positron emission tomography (PET), demonstrated disease localization to the left retrobulbar space, with no evidence of systemic involvement.

The patient initially underwent involved-site radiation therapy (ISRT) to the left orbit (13 fractions, June 2025). Concurrent antimicrobial therapy with doxycycline was administered empirically for two months. However, this intervention did not result in clinical or radiological improvement. Subsequently, the patient received four weekly infusions of rituximab (375 mg/m^2^) in September 2025.

**Figure 5:**
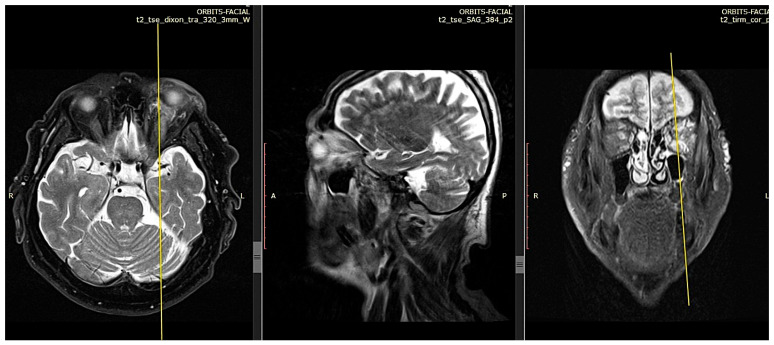
MRI, T2-weighted images from axial, sagittal, and coronal perspectives 8 months after initial treatment. Generalized thickening and deterioration of edema of all the extraocular muscles, mostly evident in the lower rectus muscle.

Both imaging studies (magnetic resonance imaging of the orbits and PET-computed tomography) and clinical examination demonstrated resistance to rituximab monotherapy. Specifically, MRI performed 8 months after the biopsy indicated persistent edema in the extraocular muscles and orbital fat, both intraconally and extraconally, with extension to the eyelids and the overlying skin of the left cheek. The optic chiasm remained unremarkable (Figure 5).

Consequently, in December 2025, the patient was initiated on systemic chemoimmunotherapy with rituximab and bendamustine, with a planned treatment course of six cycles. To date, the patient has completed two cycles of rituximab–bendamustine. Early clinical response has been documented, with a notable reduction in lower eyelid edema and relative resolution of diplopia.

## 3. Discussion

Extranodal marginal zone lymphoma (EMZL) is presented as an unusual form of B-cell lymphoma that can affect extranodal sites. Even though the ocular region is rarely affected, the extranodal marginal zone lymphoma type constitutes the most prevalent subtype of non-Hodgkin lymphoma in this area. Other less frequent types include follicular lymphoma and diffuse large B-cell lymphoma [6,12]. The most prevalent site affected in the ocular region is the ocular adnexa [6,10,13]. In our case, the lymphoma affected the extraocular muscles, presenting with edema and restricted eye movement. Isolated extraocular muscle involvement of MALT lymphoma represents an exceptionally rare presentation compared to the more prevalent manifestations affecting other ocular adnexal structures [10].

Thorough histopathological analysis is critical for the confirmation of the diagnosis due to the fact that imaging findings may be non-specific. A high-precision biopsy technique may be facilitated by using the navigational system as a supportive device tool. Thus, accurate diagnosis is enabled, preserving the surrounding delicate tissues from damage [14]. Lesions affecting the inferior orbital department may be easily accessible by means of augmented reality navigational systems [15,16]. This enables surgeons to focus on the operative field; precision and reduced complications are facilitated in complex ocular procedures by overlaying key information directly into the surgeons’ visual field [16].

Certain cases of localized EMZL, associated with chronic bacterial infections such as *Chlamydia psittaci*, may respond to antibiotic therapy alone, entering into remission and thus curing the disease. Nevertheless, the efficacy of antibiotics alone is largely dependent on the identification and eradication of a specific infective agent [5]. Alternative treatment modalities may be necessitated in cases where an infectious etiology cannot be established.

Radiotherapy is regarded as the gold-standard, first-line treatment modality for localized extranodal marginal zone lymphoma of the ocular adnexa [6,13]. The individualization of treatment, depending on the affected site and the disease extent, is highly recommended [12]. In a systematic review of 25 studies, radiotherapy demonstrated high complete response rates (often exceeding 90%) and low local recurrence rates (typically under 10%) for localized extranodal marginal zone lymphoma of the ocular adnexa [6,13]. Indicated doses ranged from 24 to 30.6 Gy, balancing efficacy with minimal toxicity to adjacent structures [6]. The use of lens shielding is recommended in order to reduce the risk of cataract formation. The use of precise radiation doses via intensity-modulated radiation therapy (IMRT) offers many advantages and minimizes the exposure to adjacent critical structures, namely, the contralateral orbit, lens, and lacrimal gland.

The use of chemotherapy is proposed for advanced-stage or disseminated extranodal marginal zone lymphoma, followed by cases where anticipating substantial visual impairment is anticipated [6,12]. The chemotherapy agents employed involve rituximab, usually combined with cyclophosphamide, doxorubicin, vincristine, and prednisone. The above combinations have demonstrated significant efficacy in achieving remission and improving patient results [12]. In addition, the IELSG-19 trial demonstrated that the combination of rituximab and chlorambucil was superior to monotherapy with either agent alone, yielding substantially improved progression-free survival and response rates [6,12].

For patients not responding to conventional treatments or experiencing disease recurrence, novel therapeutic approaches have been proposed. These include the use of targeted therapies and immunotherapies, such as Bruton’s tyrosine kinase inhibitors or programmed death-1 blockade, offering promising avenues [17]. Nevertheless, further research is essential in order to determine optimal integration of novel agents into existing treatment modalities for EMZL, especially in cases involving isolated extraocular muscle involvement [12].

A multidisciplinary approach is essential, integrating insights from oral and maxillofacial surgeons, ophthalmologists, oncologists, and hematologists. Thus, optimal therapeutic strategies are tailored for each patient. Furthermore, taking into account the rarity of isolated extraocular muscle involvement in orbital lymphoma, this case report highlights the diagnostic challenges and the need for a comprehensive, multidisciplinary approach [10].

The pathology report is also critical, particularly due to the fact that substantial raw muscle material enables comprehensive histopathological examination and immunohistochemical staining. These techniques facilitate the diagnosis of EMZL despite the surgical challenges of limited orbital tissue availability. Raw muscle biopsies are essential for the accurate diagnosis of EMZL [5,18]. Such detailed analysis is paramount in order to differentiate EMZL from other orbital inflammatory conditions that can mimic its clinical presentation. Ailments such as chronic myositis, inflammatory pseudotumor of the extraocular muscles, or infections may present similarly and are included in the differential diagnosis [5]. Therefore, a close collaboration between clinicians and pathologists is paramount when ensuring that adequate samples are obtained and processed to facilitate accurate diagnosis and guide appropriate treatment strategies [18].

## 4. Conclusions

Extranodal marginal zone lymphoma (EMZL) involving the ocular region is uncommon, and isolated involvement of the extraocular muscles represents an exceptionally rare presentation. This case underscores the diagnostic difficulties posed by its clinical and imaging resemblance to more common inflammatory conditions, potentially leading to delayed diagnosis. Effective management requires a multidisciplinary approach involving oral and maxillofacial surgeons, ophthalmologists, hematologists, oncologists, and pathologists. The integration of advanced technologies, such as image-guided navigation systems, facilitates precise biopsy acquisition from delicate orbital tissues, enabling accurate histopathological confirmation and the prompt initiation of the appropriate therapy. Early clinical suspicion, combined with technological adjuncts and close interdisciplinary collaboration, is essential for achieving favorable outcomes in patients with this rare malignancy. Long-term follow-up is recommended to monitor for potential recurrence or disease progression.

## Data Availability

The original data presented in this study are available on reasonable request from the corresponding author. The data are not publicly available due to privacy concerns.
